# Ectodermal origins of the skin-brain axis: a novel model for the developing brain, inflammation, and neurodevelopmental conditions

**DOI:** 10.1038/s41380-022-01829-8

**Published:** 2022-10-25

**Authors:** C. Jameson, K. A. Boulton, N. Silove, R. Nanan, A. J. Guastella

**Affiliations:** 1 Clinic for Autism and Neurodevelopmental Research, Brain and Mind Centre, Children’s Hospital Westmead Clinical School, Faculty of Medicine and Health, University of Sydney, Camperdown, NSW Australia; 2 Child Neurodevelopment and Mental Health Team, Brain and Mind Centre, University of Sydney, Camperdown, NSW Australia; 3 Child Development Unit, The Children’s Hospital at Westmead, Westmead, NSW Australia; 4 Charles Perkins Centre Nepean’s and Sydney Medical School Nepean, The University of Sydney, Nepean, Discipline of Paediatrics, University of Sydney, Camperdown, NSW Australia

**Keywords:** Psychology, Predictive markers

## Abstract

Early life development and its divergence is influenced by multiple genetic, neurological, and environmental factors. Atypical neurodevelopment, such as that observed in autism spectrum disorder, likely begins in early gestation during a period of entwined growth between the brain and epithelial barriers of the skin, gastrointestinal tract, and airway. This review coalesces epidemiological and neuroinflammatory evidence linking cutaneous atopic disease with both reduced skin barrier integrity and determinants of neurodivergence. We consider the shared developmental origin of epidermal and neural tissue with related genetic and environmental risk factors to evaluate potential pre- and postnatal modifiers of the skin-brain connection. Initial postnatal skin barrier integrity may provide a useful marker for both cortical integrity and meaningful subgroups of children showing early neurodevelopmental delays. It may also modify known risk factors to neurodevelopment, such as pathogen caused immune system activation. These novel insights of a skin-brain-neurodevelopment connection may advance detection and intervention opportunities.

## Introduction

Early neurodevelopmental delays are underpinned by a complex interplay between heterogeneous genetic, neurological, and environmental factors. Individuals with Autism Spectrum Disorder (ASD), for instance, show marked social delay, characterised by a continuum of difficulties during social interactions, communication, and the presence of restrictive and repetitive behaviours [1]. This neurodevelopmental divergence occurs amongst a backdrop of other physical and medical comorbidities [2, 3]. Neurocutaneous syndromes, such as tuberous sclerosis complex (TSC) and neurofibromatosis type 1 (NF1), and atopic syndromes, including atopic dermatitis, allergic rhinitis, and asthma, are all frequently reported [2, 3]. Interestingly, the global prevalence of these ‘atopic diseases’ has risen in parallel to childhood-onset ASD, recently reported to affect one in 54 children in the United States [4, 5]. This temporal synchroneity in diagnostic rates of atopic diseases and ASD has prompted investigations into the association between the two.

In this paper, we propose a novel hypothesis, that there is an antenatal link between skin and neurodevelopment, partially underpinned by the tissue’s shared ectodermal origin with common molecular factors. Additionally, we evaluate postnatal mediators of this skin-brain co-vulnerability, considering the role of epidermal keratinocytes and the cutaneous microbiota in cortical development. Accordingly, we hypothesise that skin barrier integrity may represent an accessible and novel biomarker to aid in the early detection of neurodevelopmental divergence. We further propose that skin barrier integrity may play a crucial role in mediating the relationship between environmental triggers of infection, immune processes, and neurodevelopment, with potential to reduce the impact of such environmental triggers by improving skin barrier integrity.

## Atopic comorbidities in ASD

Accumulating evidence supports an association between atopic dermatitis (AD) and neurodevelopmental divergence. This chronic, inflammatory disease is the most common paediatric skin condition in developed countries, characterised by dry and scaly cutaneous lesions, itch, pain and oozing, that manifest during the first year of life in 60% of those affected [6–8]. Interestingly, AD’s perceived link with psychopathology has been long appreciated, referred to as “neuro-dermatitis” in the early 1900s and a “psycho-somatic” disorder in the 1950s [9]. As anticipated, the observed association between AD and neurodevelopmental divergence has been extensively investigated, yet scant attention has been directed towards understanding its underlying mechanistic basis.

Population-based epidemiological studies consistently support a link between atopic eczema and ASD. Using the 2007 U.S. National Survey of Children’s Health, for instance, Yaghmai et al. [10] reported eczematic children to be 2.73 times more likely to meet the criteria for ASD, relative to their non-atopic peers (Table 1). Data from Taiwan’s National Health Insurance Database similarly indicated that atopic diseases likely precede and predict ASD onset, with 118 atopic children diagnosed with ASD at follow-up, compared to only 12 non-atopic participants [3]. Notably, ASD risk appeared to further increase alongside the number and severity of reported atopic comorbidities [3, 7].

**Table 1:** Summary of study characteristics.

Study ID, ref.	Study design	Sample size	Age (years)	Main findings	Limitations
Bakkaloglu et al. [73]	Case–control study	*N* = 71	2–4	7/30 autistic children and 7/39 age and sex-matched controls reported allergic symptoms (*p* = ns).	A small sample size of 32 children with autism and 39 controls.
Chen et al. [3]	Longitudinal follow-up study	*N* = 21 756	0–3	Early childhood atopic disease increased the risk of ASD (HR 3.40, 95% CI: 1.95–5.93). HR of ASD increased with the number of atopic comorbidities. 1: HR 2.14 (95% CI: 0.90–5.11) 2: HR: 2.70 (95% CI: 1.44–5.05) 3: HR: 4.08 (95% CI: 2.24–7.43) 4: HR: 4.29 (95% CI: 2.25–8.19)	ASD incidence was likely underestimated as the sample only included those who sought medical attention.
Gurney et al. [12]	Population-based cross-sectional study	*N* = 85 272	3–17	Atopic prevalence in ASD cohort *vs*. control Hay fever: 27% *vs*. 16.2% OR: 1.6 (95% CI: 1.1–2.4) Food allergy: 14.1% *vs*. 3.2% OR: 4.5 (95% CI: 3.0–7.0) Eczema or skin allergy: 14.9% *vs*. 9.2% OR: 1.4 (95% CI 1.0–2.1)	Unvalidated parental report of atopic manifestations and no subtype analysis of ASD.
Liao et al. [7]	Population-based case–control study	*N* = 32 956	0.08–3	HR of ASD increased with the number of eczema-related doctor visits. 1: aHR 1.15 (95% CI 1.06–1.24) 2–3: aHR 1.18 (95% CI 1.07–1.29) 4+: aHR 1.40 (95% CI 1.25–1.56)	Data may be confounded by patterns of help-seeking behaviour.
Magalhães et al. [74]	Case–control study	*N* = 45	7–18	86.6% of Asperger patients were atopic (allergic symptoms, elevated serum IgE, high eosinophil count or positive skin prick test), compared to <7% of controls	A small sample size of 15 Asperger patients, 15 atopic neurotypicals and 15 non-atopic neurotypicals
Miyazaki et al. [11]	Systematic review and meta-analysis	*N* = 10 380	2.5–18	Prevalence of asthma and allergic rhinitis were significantly increased in the ASD cohort (OR 1.69; 95% CI: 1.11–2.59 and OR 1.66; 95% CI: 1.49–1.85 respectively)	ASD diagnostic criteria varied across studies (e.g. DSM-V *vs*. ADOS *vs*. ICD-10)
Tsai et al. [75]	Systematic review and meta-analysis	*N* = 1 055 837	0–18	Prevalence of AD was significantly higher in individuals with ASD than the control group OR: 1.485 (95% CI: 1.20–1.83)	Heterogeneity in diagnostic criteria, age, race and disease severity across studies.
Xu et al. [5]	Population-based, cross-sectional study	*N* = 199 520	3–17	Children with ASD were more likely to report food allergy (11.25% *vs*. 4.25%), respiratory allergy (18.73% *vs*. 12.15%) & skin allergy (16.81% *vs*. 9.84%) than a non-autistic comparison group.	Retrospectively collected self-reported data may have enabled misreporting due to recall bias
Yaghmai et al. [10]	Cross-sectional study	*N* = 92 642	0–17	Prevalence of ASD was significantly increased in children with eczema (2.19%) compared to non-autistic controls (0.89%). OR ASD: 2.73 (95% CI 1.94–3.84)	Parental reporting subject to misclassification bias
Zerbo et al. [76]	Case–control study	*N* = 33 390	3–26	Allergies were more frequently diagnosed in ASD cohort (20.6%) *vs*. controls (17.7%) OR: 1.22 (95% CI 1.13–1.31)	Did not account for heterogeneous ASD subtypes.

Given that AD often marks the onset of the “atopic march”, facilitating subsequent allergic sensitisation, various atopic manifestations should co-occur in neurodiverse populations. Accordingly, a meta-analysis including 10 380 children [11] found increased rates of asthma and allergic rhinitis in participants with ASD relative to non-autistic populations. Similarly, using the 2004 U.S. National Survey of Children’s Health, Gurney et al. [12] observed eczema, hay fever and food allergy to be significantly more prevalent amongst 483 children with autism compared to their neurotypical peers. Additionally, analysing data from the U.S. National Health Interview Scheme, Xu et al. [5] reported across a large and multi-racial sample that autistic children were more likely to experience food allergy (11.25% vs. 4.25%), respiratory allergy (18.73% vs. 12.15%) and skin allergy (16.81% vs. 9.84%) relative to matched controls. More recently, one recent study in children with ASD suggested that the presence of comorbid atopic conditions was associated with more severe ASD symptoms, with the prensence of atopic diseases more than doubling the likelihood of severe ASD symptoms and severe social impairment scores  [13].

## Antenatal parallels between cortical and epidermal development

Overall, whilst the positive association between atopic disorders and ASD is well-established, the biological basis of this association has not been elucidated. Here, we consider the potential prenatal genesis of this link, reviewing the shared in utero development of skin and neural tissue alongside common genetic susceptibility variants.

Gastrulation is a fundamental process in early embryonic development, beginning at three post-conception weeks (PCW) to induce the formation of three distinct cell layers: the mesoderm, endoderm, and ectoderm [14]. Over the following 5 weeks, these primary germ layers undergo organogenesis, differentiating into the body’s organs [14]. Importantly, the brain, skin and skin appendages develop in synchronisation, all originating from the embryonic ectoderm [15]. Accordingly, from 3 to 4 PCW, the ventral ectoderm differentiates into a monolayer epidermis whilst the dorsal ectoderm becomes the neuroectoderm, from which early derivatives of the brain develop [14]. It is therefore plausible that early deviations to neural development may influence patterns of epidermal differentiation.

Preliminary evidence supporting this hypothesis can be derived from a study [16] investigating *Xenopus laevis* embryonic development. The researchers demonstrated that upregulated Cdc2-like kinase 2 (Clk2), found in neural tissue to augment signalling pathways regulating organogenesis, promotes neural plate growth at the expense of epidermal development. Their findings revealed that Clk2 overexpression both induced the neural gene markers sip1 and sox3 whilst reducing epidermal keratin expression compared to controls. This is striking in the context of neurodevelopmental disorders, such as ASD, as elevated Clk2 has been linked to synaptic disruption in Shank3-deficient neurons in mouse models of ASD [17]. Further, the Clk2-induced neural plate expansion observed during *Xenopus* embryogenesis holds similarities with the prenatal cortical overgrowth often observed in autistic children [18]. The transferability of these findings is also strengthened by Clk2 being the most abundantly expressed Clk family member in the mammalian brain [16]. Previous literature, therefore, indicates that abnormal neural development can alter epidermal differentiation in utero.

Further supporting a fundamental link between skin and neurodevelopment are studies investigating ectoderm-derived minor physical anomalies (MPAs) in neurodivergent populations. MPAs are subtle phenotypic deviations to the craniofacial or limb regions, observed to occur significantly more frequently in those with neurodevelopmental delay (up to 60%), such as that seen in fetal alcohol syndrome, attention-deficit/hyperactivity disorder (ADHD) and ASD [19–21]. Consequently, prior literature has posited MPAs to represent surface biomarkers that may reflect atypical neurodevelopment arising during the first trimester of pregnancy [19, 20]. In support of this hypothesis, Miles et al. [22] reported that MPAs strongly correlate with structural brain anomalies, with 19 adults showing MPAs being twice as likely (29%) to record abnormal MRIs relative to 51 adults without morphological deviations (14%). Their findings were later replicated in children, where inter-orbital MPAs were observed to positively correlate with bilateral amygdalae volume in 36 children with ASD [19]. Overall, evidence correlating ectodermal-derived MPAs with neurodevelopmental divergence further supports a link between cortical and epidermal development.

Moreover, if an ectodermal link exists between skin and neurodevelopment, epidermal abnormalities should co-occur with various neurodevelopmental conditions. Using data from the Danish National Patient Registry, Vittrup et al. [23] reported that eczema was significantly associated with ADHD in 157 113 children (Table 2). This coincides with data from the U.S. National Health Interview Survey spanning 2008–2018, observing in 109 483 children that AD was associated with caregiver-reported developmental delay and ADHD [24]. These findings further align with an earlier cross-sectional study, which found that 36% of 639 children showing language delay and problems in internalisation and externalisation symptoms presented with comorbid AD [25]. Interestingly, the association between neurodevelopmental delay and altered skin development appears to persist beyond paediatric populations. In an adult prospective study, for instance, 66.8% of 366 AD patients self-reported at least one symptom of cognitive dysfunction, with 10.6% showing moderate to severe cognitive impairments, determined by their PROMIS Cognitive Function scores [26].

**Table 2:** Summary of study characteristics.

Study ID, ref.	Study design	Sample size	Age (years)	Main findings	Limitations
Jackson-Cowan et al. [24]	Population-based cross-sectional study	*N* = 109 483	2–17	Childhood AD was significantly associated with developmental delay (aOR: 1.54; 95% CI: 1.40–1.40) (*p* < 0.001) and ADD/ADHD (aOR: 1.31; 95% CI: 1.20–1.42) (*p* < 0.001)	Did not control for sedating medications that may have influenced cognitive function (e.g. antihistamines)
Kandelaki et al. [25]	Cross-sectional study	*N* = 639	5–6	36% of children with language delays and problems in internalisation and externalisation reported comorbid AD	Cross-sectional design prevented the inclusion of follow-up data
Silverberg et al. [26]	Prospective study	Baseline: *N* = 366 Follow-up: *N* = 245	18–88	Baseline: 66.8% self-reported ≥1 symptom of cognitive dysfunction (e.g. slowed thinking, difficulty concentrating) Follow-up: 5.4% showed moderate and 5.2% showed severe PROMIS cognitive function T-scores	The baseline cognitive function questionnaire was self-administered and may be subject to recall bias
Vittrup et al. [23]	Population-based longitudinal study	157 113	0–18	Childhood AD was significantly associated with ADHD HR: 1.89 (95% CI: 1.56–2.29)	AD severity was classified based on the proxy of medication use, allowing for misclassification bias

## Molecular factors common to brain and skin development

The association between skin and neural development is further strengthened by common molecular factors, including brain-derived neurotrophic factor (BDNF) and filaggrin, which appear abnormally expressed in both ASD and AD.

### Brain-derived neurotrophic factor

BDNF is an activity-dependent neurotrophin regulating synaptic plasticity and the development of cortical connections [27]. BDNF acts via the pan-neurotrophin receptor (p75^NTR^), to which all mature neurotrophins bind, or the tropomyosin receptor kinase B (TrkB), to which it shows high affinity and specificity [28]. Numerous studies have detected elevated serum and plasma concentrations of BDNF in children with autism, compared to non-autistic controls [27, 29, 30]. BDNF has therefore been proposed to contribute to altered neurodevelopment in ASD pathology. In neurotypical individuals, for instance, BDNF increases pre-synaptic glutamate release and post-synaptically augments NMDA receptors, inducing excitatory activity that is dampened by neighbouring receptors [29]. In those with ASD, however, this negative regulation appears to be impaired by synaptic dysfunction, provoking cytotoxicity associated with neurodevelopmental divergence [27].

Interestingly, BDNF may also mediate the interaction between immune cells and neurons in chronic atopic conditions [28]. For instance, elevated BDNF has been observed in bronchoalveolar lavage samples of 8 male asthmatics [31] and the nasal mucosa of 9 allergic rhinitis patients following allergen exposure [32]. Numerous studies have also identified significantly increased serum BDNF concentration in eczematic children relative to non-atopic participants [28, 33]. Notably, in those with AD, BDNF appears to be expressed by cutaneous eosinophils, which promote axonal outgrowth and neurite branching in dorsal root ganglion neurons when stimulated by a plate-activating factor [34]. Further, BDNF has been observed to elicit chemotaxis and halt eosinophil apoptosis exclusively in AD patients [33]. Taken together, these findings support BDNF’s role in provoking morphological and pro-inflammatory changes in the cutaneous neurons of those with AD [34]. BDNF’s neurotrophic and immunomodulatory activities may therefore increase susceptibility to both atypical neurodevelopment and AD-associated cutaneous inflammation.

### Filaggrin

Additionally, the *FLG* gene has garnered attention, with common loss-of-function mutations determined as susceptibility variants for both ASD and AD [35–37]. *FLG* is part of the epidermal differentiation gene complex encoding the precursor for filaggrin [6]. Mature filaggrin units themselves are fundamental to the stratum corneum, aggregating keratin intermediary filaments and regulating terminal differentiation via disulphide bond formation [37]. When proteolysed, filaggrin also assists in epidermal water retention by contributing to the stratum corneum’s Natural Moisturising Factors (NMF) [37]. Interestingly, *FLG* is located on chromosome 1q21, a region of the genome where deletion and duplication carriers have been observed to be at a higher risk for ASD [38]. For example, a large-scale neuroimaging study of over 37 000 participants found that brain structural abnormalities detected in 1q21.1 variant carriers overlapped with cortical deviations often observed in those with ASD [38]. Recently, *FLG* itself was also identified as a possible genetic risk factor for ASD [35, 36]. For instance, Shi et al.’s [36] genome-wide association study found seven candidate genes shared by two siblings with autism, one of which was *FLG* with two deleterious mutations. Similarly, Chang et al. [35] later identified *FLG* as one of seven pathogenic variants found in four probands, observing a p.E2322X mutations in the *FLG2* gene.

Unsurprisingly, *FLG* loss-of-function variants have been long-identified as major risk factors for AD in children [35–37]. Flohr et al. [6] for instance, observed in 88 Caucasian infants that those carrying a common *FLG* mutation were four times more likely to experience eczema by 3 months of age than those without the variant. This association between *FLG* mutations and xerosis appears to be underpinned by a reduction in NMF and elevations in skin pH that provoke downstream effects, including abnormal lipid transport to the stratum corneum [9].

From an evolutionary perspective, the variability profiles of genes underlying ectodermal and endodermal organ development are strikingly distinct. Whilst the genetic mechanisms regulating endoderm-derived organ development have shown a high degree of evolutionary conservation across various vertebrate classes [39], gene families coordinating ectodermal development appear to be strongly driven by adaptive evolution [40]. The human brain, for example, has evolved extensively throughout history, responding to changes in our environment and climate, as evidenced by a rapid expansion of higher-order functional networks [40, 41]. Notably, the skin has shown analogous evolutionary potential, with the loss of body hair provoking the evolution of genes regulating keratinisation and epidermal differentiation to enhance cutaneous barrier function [42]. To date, the complete repeats of *FLG* continue to show high nucleotide diversity and copy number variation across primates [43]. Interestingly, this similarity in neural and epidermal evolvability may arise from the decentralised neurological system of primitive multicellular animals, including Cnidarians, possibly marking the origins of the surface ectoderm’s link with brain development [44]. Therefore, whilst endoderm-derived organ systems remain highly conserved across evolution and taxa, the genetic basis of ectodermal development has shown remarkable adaptability, which may partially underly the basal link between skin aberrations and neurodevelopmental divergence.

## Postnatal modifiers of the skin-brain connection

### Epidermal keratinocytes

The principal link between epidermal and neurodevelopment has further been observed to persist postnatally, with the notion of a skin-brain or brain-skin axis growing in popularity. Epidermal keratinocytes appear to be central to this association, with these ‘information and sensory processing cells’ expressing numerous receptors found within the central nervous system (CNS) [44, 45]. NMDA receptors, for instance, are expressed in both epidermal keratinocytes, influencing epidermal proliferation and barrier maintenance, and the hippocampus, where their dysfunction has been associated with ASD-related pathology in mice [45, 46]. Keratinocytes also appear to release oxytocin, a fundamental hormone in behaviour and social bonding, in the presence of an ATP analogue [47]. This is significant in the context of ASD, where oxytocin may improve social cognition and development [48]. Notably, epidermal cells also seem to have the capacity to convert into neural cells, with Tenorio-Mina et al. [49] transdifferentiating human keratinocytes into neural progenitors in vitro that produce neuronal proteins when transplanted into a developing rat brain. Taken together, prior research suggests that epidermal keratinocytes may house sensory systems and secrete bioactive molecules able to postnatally influence neural functioning.

### HPA-axis mediated brain-skin crosstalk

Persistent neuro-epidermal communication further appears to be coordinated by the sympathetic nervous system (SNS), whose sensory neuron cell bodies occupy dorsal root ganglia innervating both the skin and CNS and the hypothalamic-pituitary-adrenal (HPA)-axis [18, 50]. Interestingly, mammalian epidermal keratinocytes express all HPA-axis components, which function to regulate cutaneous anti-microbial defence [18, 51]. Consequently, environmental factors disrupting axis activity, including stress, have been associated with epidermal abnormalities, inducing the activation of cutaneous mast cells and corticotrophin-releasing hormone, stimulating cytokine secretion [50]. The neural modulation of epidermal functioning is additionally supported by Hunter et al. [52] observations of slowed cutaneous wound healing during periods of heightened stress, perhaps attributable to the SNS release of noradrenaline and adrenaline associated with inhibited fibroblast growth and proliferation [53]. In summary, previous literature supports a skin-brain connection modified by the HPA-axis postnatally, pointing to the epidermal barrier as possibly representing a “diagnostic window into the brain” from which simple biomarkers of neurodevelopment could be identified [50].

### Childhood inflammation

The cutaneous microbiota and postnatal skin inflammation may represent additional early life modifiers of neurodevelopment. After birth, infant skin is rapidly colonised by diverse microbiota, prompting epidermal maturation to protect against the transcutaneous invasion of external irritants [54]. In instances of skin barrier deficiency, the colonising microbiome may provoke cutaneous infection, transforming the largest organ of the body into a potential source of systemic inflammation [55]. This phenomenon has been observed in an N-WASP^CK14-Cre^ KO mouse model, where induced epidermal barrier defects were associated with increased mast cell and eosinophil influx to the dermis as well as a significantly increased serum concentration of pro-inflammatory cytokines, including interleukin (IL)-1α, IL-6 and IL-17 [55]. Interestingly, elevated IL-6 and IL-17A have similarly been detected in the plasma and serum of children with ASD, with prior research indicating that these mediators may traverse the BBB and provoke neuroinflammation in circuitry regulating developmental outcomes [56]. Upregulated inflammatory processes provoked by the cutaneous microbiota’s colonisation of a damaged epidermal barrier may therefore act as a postnatal modifier of neurodevelopment.

### Thermoregulation

To conclude our review of the postnatal skin-brain connection, we turn our attention towards the skin’s thermoregulatory function. Following the loss of fur, hominin’s capacity for thermoregulation advanced, shifting from thermal panting to high-density eccrine sweat glands [42]. In the same period, our cognitive capacity increased emphatically, spurring interest as to whether thermoregulation may influence neural development. Preliminary evidence for this hypothesis stems from recent zebrafish models, where the ambient water temperature was observed to drive persistent changes in exploratory behaviour [57]. These findings indicate that thermoregulatory mechanisms may drive intra-individual variability in vertebrate kinematics, likely by influencing neural circuits that regulate locomotor control [57]. In humans, anecdotal evidence supporting a link between thermoregulation and neurodevelopment can be derived from parental reports of improved social interaction and language ability during febrile states in autistic children [58]. Additionally, daily skin-to-skin care in preterm infants, which provides thermoregulatory stimulation, has been observed to improve children’s cognitive development and executive functioning at 10 years of age, relative to no-contact infants [59]. In summary, prior literature indicates that thermoregulation may modify neural development, providing further evidence for a fundamental skin-brain connection persisting postnatally.

## Plausible skin barrier integrity markers for neurodevelopmental divergence

### Transepidermal water loss

**Fig. 1:**
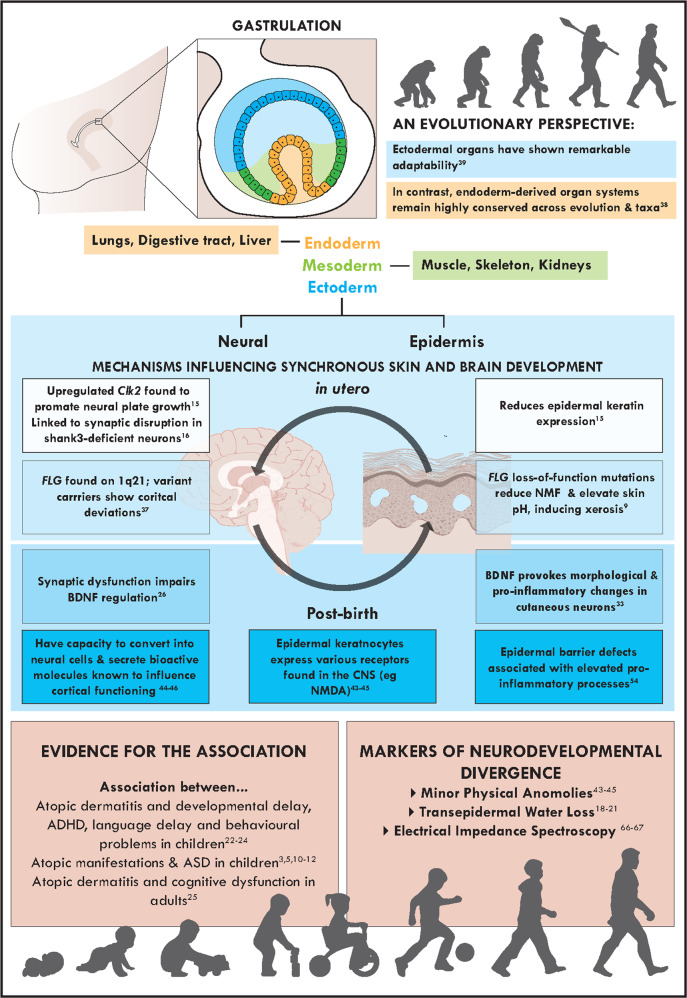
The ectoderm and neurodevelopmental divergence. This figure describes the evolutionary and developmental skin-brain connection, common molecular factors that underpin this relationship in utero and post-birth, the existing evidence for associations between atopic disease and neurodevelopmental delay, and potential early life markers that could identify neurodevelopmental divergence.

**Fig. 2:**
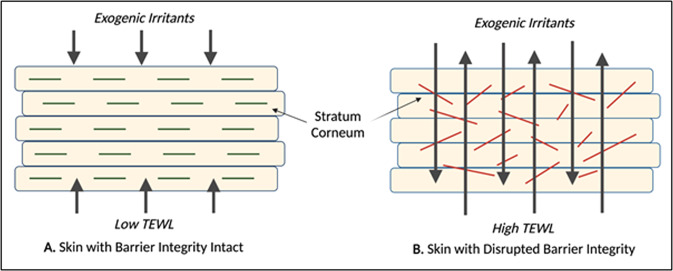
A schematic diagram showing how the integrity of the skin barrier may influence allergen penetration and TEWL measurements in (**A**) Healthy skin and (**B**) Disturbed skin.

Thus far, this paper has reviewed accumulating evidence supporting a principal link between cortical and epidermal development, underpinned by their common in utero origin and genetic susceptibility variants, that endures postnatally (see Fig. 1). Consequently, we propose that skin barrier integrity might represent an early indicator for neurodevelopmental divergence. The most widely used tool to measure epidermal barrier function in vivo is transepidermal water loss (TEWL), a non-invasive technique that calculates water’s flux density as it diffuses from the dermis and epidermis, through the stratum corneum, to the skin’s surface (Fig. 2) [60]. An elevated TEWL indicates a disrupted epidermal barrier, increasing the ease with which microbes and immunogens can penetrate the skin [60]. Given the proposed skin-brain co-vulnerability and frequent reports of cutaneous disorders in neurodiverse populations, we consider whether TEWL may serve as a novel biomarker for detecting early neurodevelopmental delay [61].

Epidermal barrier function in atopic dermatitis has been extensively investigated. For instance, Seidenari and Giusti [62] observed elevated TEWL at eczematous skin of the forehead, cheek, volar and dorsal forearm, and abdomen of 100 children with AD compared to 21 healthy participants (Table 3). Their findings were later replicated across a spectrum of measurement sites, with significant increases in TEWL also detected at lesions of the postauricle, thigh, and popliteal fossa of 25 AD patients [63]. Further, this association remains perceptible in infant cohorts [64], with elevated TEWL at the non-lesional upper arm being significantly associated with an eczema diagnosis at follow-up. Increased TEWL, therefore, appears to precede and predict AD onset, and is indicative of widespread epidermal barrier dysfunction, with Montero-Vilchez et al. [65] measuring heightened TEWL at the non-lesional skin of eczema patients relative to non-atopic controls.

**Table 3:** Summary of study characteristics.

Study ID, ref.	Study design	Sample size	Age (years)	Main findings	Limitations
Berents et al. [77]	Population-based cohort study	*N* = 116	1.2–13.4	TEWL > 9.33 g/m^2^h at visit 1 was significantly associated with a diagnosis of AD at visit 2 (*p* = 0.03) AD OR: 3.32 (95% CI: 1.15–9.60)	TEWL was measured by multiple clinicians at two medical services across a range of humidity
Kim et al. [63]	Case–control study	*N* = 50	12–39	The mean TEWL value measured across AD participants was significantly greater than that of age-matched healthy controls at all evaluation sites (*postauricle, forearm, abdomen, thigh, and popliteal fossa*)	TEWL was measured using an open-chamber device (vulnerable to ambient airflow)
Montero-Vilchez et al. [65]	Cross-sectional study	*N* = 130	X̅ = 32.05	Mean TEWL was elevated at the uninvolved skin of AD patients (13.15 g/m^2^h) compared to healthy controls (11.60 g/m^2^h)	Cross-sectional design prevented participant follow-up
Seidenari and Guisti [62]	Cross-sectional study	*N* = 121	3–12	TEWL was increased at the eczematous lesions of children with AD compared to control subjects (*p* < 0.05)	TEWL was measured using an open-chamber device (vulnerable to ambient airflow)
Shin et al. [66]	Randomised controlled trial	*N* = 20 first-generation *N* = 100 pups	Neo-natal	Cutaneous scaling was observed in VPA mice. TEWL levels measured 4 days post-partum were elevated in VPA mice compared to controls	The unique physiology of the human brain limits the transferability of mouse model findings
Shin et al. [66]	Randomly selected cohort study	*N* = 25	X̅ = 19	Abnormalities in skin barrier permeability and skin hydration were observed in the ASD cohort, yet these did not reach statistical significance (*p* = 0.2)	A small sample size (*n* = 25) Differences in TEWL may be more difficult to discern in adolescent populations (influenced by age-associated changes in the stratum corneum)

Investigations into skin barrier integrity in neurodivergent children, however, are scarce. In animals, Shin et al. [66] conducted a randomised controlled trial to evaluate epidermal integrity in a valproic acid (VPA)-induced mouse model of autism. As anticipated, the VPA-exposed neo-natal mice displayed both neural and cutaneous structural abnormalities, displaying vacuolisation exclusively in the brain and outer nucleated layer of the epidermis. Interestingly, the VPA mice additionally showed significantly elevated TEWL measurements, taken 4 days post-partum, relative to the controls. Whilst this rodent model offers preliminary insight into the role of cutaneous permeability in ASD, the unique physiology of the human brain limits the transferability of their findings. Shin et al. [67] also investigated skin barrier function in a cohort of 25 adolescents with ASD. In line with their mouse model findings, TEWL appeared likely elevated in individuals with ASD, however, differences did not reach statistical significance in this small sample size, with adolescent-associated corneocyte flattening and heightened sebum secretion also possibly reducing the ability to discern differences between groups [60].

### Electrical impedance spectroscopy

Electrical impedance spectroscopy (EIS) has recently emerged as a promising alternative measurement of skin barrier function. EIS is a non-invasive indicator of epidermal integrity, rapidly measuring the skin’s resistance to the flow of alternating imperceptible currents [67, 68]. Considering the likely differences in cell size and orientation between typical and abnormal skin, EIS has been suggested as a useful proxy for disease status [67, 68]. Currently, EIS has been observed to successfully differentiate between healthy, lesional and non-lesional skin in adults with AD, with these readings inversely correlating to TEWL [68]. To date, no study has measured EIS in neurodevelopmentally diverse populations. Preliminary evidence from eczematic patients, however, has shown EIS to negatively correlate with serum proteins associated with the inflammatory response, indicating that upregulated inflammatory processes, as observed in ASD, may be associated with weaker barrier function [69]. Of particular interest, EIS measured at the non-lesional skin of adults with AD was found to correlate with the number of tandem repeats in the *FLG* gene, an established susceptibility variant for both ASD and skin abnormalities [38, 68]. EIS may therefore represent an alternate clinical measure of skin barrier integrity that may function as a surrogate for neurodevelopmental divergence.

## Tactile sensitivity

Sensory processing atypicality is increasingly recognised as an important diagnostic and common feature of ASD [69], which may include both hyper- and hypo-sensitivity to touch [70]. The current biological mechanisms proposed to underpin this touch-sensitivity skin-brain connection is largely independent to what has been discussed in this review [70–72]. While it is beyond the scope of this review to speculate about any commonalities, future research is required to understand common biological mechanisms that could contribute to both the development of skin barrier integrity and adaptive tactile sensory processing.

## Conclusion

In summary, this paper reviewed epidemiological and clinical evidence supporting a fundamental link between skin and neurodevelopment, antenatally mediated by a shared developmental origin and genetic susceptibility variants. Moreover, we consider how the skin, being the body’s largest organ, is a fundamental barrier to diseases, viruses, and infections, and may therefore have a neuroprotective effect by reducing systemic inflammation associated with atypical neurodevelopment. Subsequent to evaluating postnatal modifiers of this skin-brain co-vulnerability, we propose that skin barrier integrity might represent an early indicator of atypical neurodevelopment. Our approach addresses the need for easily accessible clinical tools that support the detection of neural diversity and offers plausible biological and environmental contributors to early life development.
